# Relationship Between Peripheral Arterial Diseases and Obstructive Sleep Apnea: A Systematic Review

**DOI:** 10.7759/cureus.35550

**Published:** 2023-02-27

**Authors:** Sultan AlSheikh

**Affiliations:** 1 Division of Vascular Surgery, Department of Surgery, College of Medicine, King Saud University, Riyadh, SAU

**Keywords:** apnea-hypopnea index, ankle-brachial index, sleep apnea, peripheral arterial disease, obstructive sleep apnea

## Abstract

Although the effects of obstructive sleep apnea (OSA) on the cardiovascular outcomes of coronary artery disease (CAD) have been well-established, its significance on the occurrence of peripheral arterial disease (PAD) remains debatable. Prompt diagnosis and treatment of OSA would help reduce cardiovascular comorbidities. Our study aimed to assess the relationship between OSA and PAD and report any statistical association between the two conditions. Here, we investigated the prevalence and association of OSA in PAD based on related articles from PubMed, Embase, and the Cochrane Library. All databases were systematically searched from January 2000 to December 2020. A total of 238 articles deemed relevant were assessed for eligibility, of which seven articles were selected for the systematic review. Seven prospective cohorts were qualified for inclusion, which included 26,881 male and 34,403 female patients (N = 61,284). The retrieved articles described OSA severity based on the apnea-hypopnea index and reported increased OSA prevalence in PAD patients. The Epworth sleepiness scale showed no association between OSA severity, poor ankle-brachial index values, and increased daytime sleepiness. The prevalence of OSA increased in patients with PAD. Further research and prospective clinical trials are required to establish strong associations between OSA and PAD to make appropriate changes in patient management algorithms and improve their outcomes.

## Introduction and background

Obstructive sleep apnea (OSA), a chronic sleep-related breathing difficulty due to recurrent upper airway collapse, is characterized by snoring, and daytime sleepiness, which are the main manifestations [1]. Available evidence suggests that OSA is often underdiagnosed, and its prevalence varies between 9% and 38% in the general adult population. The senior population, male patients, and patients with obesity are at higher risk [2]. Currently, the population-based Swiss HypnoLaus study recorded the highest prevalence estimate of 72% [3]. Still, variation in its prevalence has been observed due to the study period, cohort size, participants' age, diagnosis methods, and scoring criteria used.

Further, the implementation of new recording technologies improved scoring criteria, an increase in the prevalence of obesity and longer life expectancy in addition to increased respiratory rates, sleep fragmentation, and gaseous exchange disorders have made OSA an increasingly prevalent public health issue, either or both partial and total upper airway obstruction during sleep [1], enabling airway narrowing (hypopnea) or occlusion (apnea). There are variations in the manifestations between adults and children concerning pathophysiology, clinical characteristics, and treatment [4]. Sleep disorders are common issues that jeopardize a patient's quality of life and overall health. The effect of OSA on children with respect to their mood, expressions, language, cognitive abilities, school performance, and visual perception must be considered [5-7]. Given the variety of etiological determinants and consequences, the diagnosis and management of sleep disorders demand interdisciplinary efforts [8,9]. OSA may also impair the cardiovascular system, with numerous studies suggesting that OSA is linked to poor cardiovascular findings such as coronary artery disease (CAD), stroke, atrial fibrillation, and myocardial damage [10,11].

Furthermore, atherogenesis could occur due to repetitive intermittent hypoxemia on the bases of inflammation, oxidative stress, dyslipidemia, etc. [12]. However, despite the reported association between OSA severity and increased myocardial infarction, stroke, and death risks due to cardiovascular diseases (CVDs), the prevalence and significance of OSA on peripheral artery disease (PAD) occurrence are poorly understood [13]. A recent study reported that at least 48% of PAD cases also had OSA, in whom OSA was mild in 28% of patients, moderate in 15%, and severe in 6% of the cases [14]. PAD, a poor prognostic factor for CVD and a marker of atherosclerosis severity, has also been reported to possess similar risk factors as OSA, such as diabetes, obesity, hypertension, etc. [15]. Moreover, severe OSA accompanies rapid eye movement (REM) sleep and has also been related to a higher incidence of cardiometabolic complications in people with existing cardiac comorbidities [16]. Thus, the co-existence of OSA and PAD could aggravate the progression of both disorders, worsen patients' prognoses, and negatively impact their treatment outcomes. We systematically reviewed the relationship between PAD and OSA to shed more light on this topic.

## Review

Materials and methods

Protocol and Reporting

**Figure 1:**
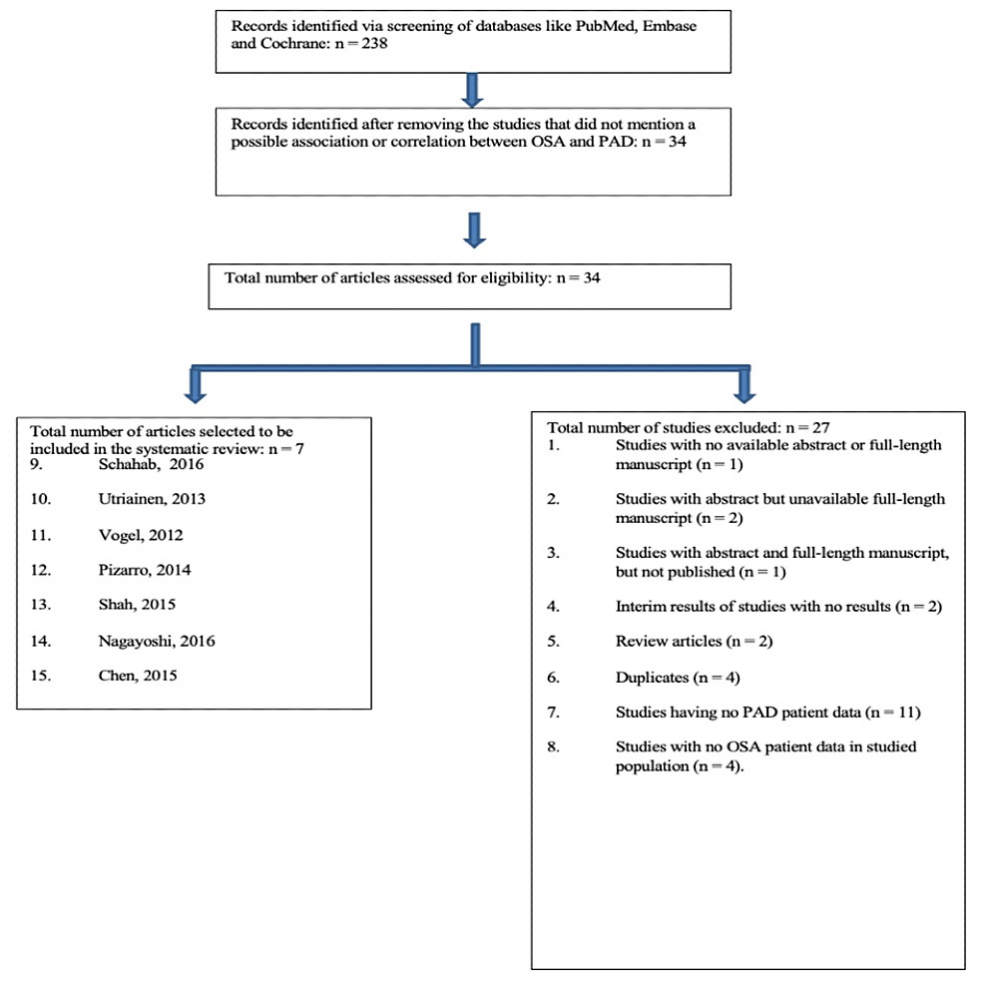
PRISMA flowchart * n, number; OSA, obstructive sleep apnea; PAD; peripheral arterial disease; PRISMA: Preferred Reporting Items for Systematic Reviews and Meta-Analyses

This present systematic review was performed following the Preferred Reporting Items for Systematic Reviews and Meta-Analyses (PRISMA) guidelines. The PRISMA flowchart (Figure 1) displays the methodology in terms of the search strategy for study inclusion and exclusion and the reasons for including and excluding studies.

Selection Criteria

We focused on articles evaluating the association between OSA and PAD based on the following inclusion criteria:

1. evaluated patients with OSA and PAD;

2. assessed the relationship between OSA and PAD;

3. retrospective studies that have evaluated the presence of PAD in patients diagnosed with OSA.

Studies were excluded if they had:

1. assessed the presence of cardiovascular conditions other than PAD in patients with OSA;

2. assessed the relationship between PAD and respiratory conditions other than OSA;

3. incomplete data with respect to the methods and results;

4. studies with no available abstract or full-length manuscript;

5. interim results of studies with no final results;

6. article with no subjective data; these included case reports, media talks, and correspondence;

7. articles with no objective results;

8. studies having no PAD patient data;

9. studies with no OSA patient data in the studied population.

Finally, studies that were reported in the form of abstracts, duplicates, and unpublished articles were not eligible for inclusion. For screening, all relevant articles in English were chosen.

Data Retrieval and Methodology

The search nomenclature was based on the systematic review protocol. The search strategy involved using search terms regarding the presence of PAD in patients with OSA. The terms that describe PAD were used with the Boolean operator OR so that related articles are not missed. The search for related articles consisted of free text and controlled language searches. The free text search consisted of words such as “peripheral arterial diseases,” “veno occlusive diseases,” “obstructive sleep apnea,” and “Chronic arterial diseases.” We also included the following Medical Subject Headings terms: “Relationship between PAD and OSA,” “Association between the presence of obstructive sleep apnea and peripheral arterial disease,” and “Chronic veno occlusive diseases and its relationship with the presence of obstructive sleep apnea.”

Independent reviewers screened for potentially eligible literature published on PubMed, Cochrane Library, and Embase databases from January 1, 2000, to December 31, 2020. Their title and abstract were independently evaluated, and the results with the selection criteria and qualified studies were short-listed. Furthermore, the original text of the selected studies was retrieved and reviewed in terms of research content, which included patient outcomes following coiling and clipping for aneurysm repair. Lastly, the references of these manuscripts were also reviewed to identify potentially eligible studies.

Data Extraction and Risk-of-Bias Assessment

Two reviewers obtained information separately and recorded it in a customized data extraction sheet. Disagreements were resolved through mutual discussion or consultation with a third reviewer. The original studies’ contents were evaluated for inclusion in the initial stage. Key contents based on the relationship between PAD and OSA were retrieved and assessed. Two separate reviewers independently evaluated the risk of bias in each study.

Statistical Analysis

Standard mean differences (SMD) or mean differences (MD) were used to determine the relationship between each study’s outcomes with the Review Manager software version 5 (RevMan, Copenhagen: The Nordic Cochrane Centre, The Cochrane Collaboration, 2014). A meta-analysis was performed to obtain the absolute values and combined measures of the effect of each primary outcome based on the expected heterogeneity between trials. The standardized mean difference with 95% confidence intervals (CI) will be used. P < 0.05 was used to show significant differences between comparisons. All the statistical analyses were conducted with the Stata/SE software (v14.0; StataCorp, College Station, Texas).

Results

Baseline Characteristics

A total of seven prospective clinical studies were eligible for this meta-analysis, including 26,881 male and 34,403 female patients (N = 61,284) (Table 1). Most patients could be considered elderly, with a mean age of 59.6 ± 9.1 to 71.1 ± 9.8 years. Five studies reported data on mean, body mass index (BMI) was reported in five studies, comprising 13,964 patients and ranging between 26 and 32 kg/m^2^. We also observed that PAD patients had a significantly higher prevalence of hypertension, diabetes mellitus (DM), smoking, and hyperlipidemia.

**Table 1:** Baseline characteristics N: number; SD: standard deviation; BMI: body mass index; kg/m^2^: kilogram per square meter; NA: not applicable; PAD: peripheral arterial disease

Studies	N (number of subjects)	Age in years (mean ± SD)	BMI (kg/m^2^)	Hypertension	Diabetes Mellitus	Smoking	Hyperlipidemia
Vogel et al., 2012 [17]	52	62 ± 11	NA	NA	NA	NA	NA
Schahab et al., 2016 [18]	59	71.1 ± 9.8	27.7 ± 4.3	96.6%	33.9%	NA	57.6%
Pizarro et al., 2014 [19]	91	63.9 ± 10.8	26.8 ± 3.9	NA	NA	NA	NA
Shah et al., 2015 [20]	8367	60.7 ± 7.1	29.8 ± 4.2	PAD: 63.9% Control: 41.3%	PAD: 44.7% Control: 26.5%	PAD: 58.2% Control: 43.5%	PAD: 50.1% Control: 44.3%
Utriainen et al., 2013 [21]	82	67 ± 9	27 ± 4.1	76%	43%	38%	54%
Nagayoshi et al., 2016 [22]	5365	59.6 ± 9.1	32.1 ± 6.2	51.6%	12.1%	NA	NA
Chen et al., 2015 [23]	47268	60 ± 15.3	NA	PAD: 52.2% Control: 39.4%	PAD: 30.8% Control: 19.5%	NA	PAD: 26.5% Control: 17.9%

OSA

Diagnosed or suspected OSA was present in 3,606 (5.88%) patients. Polysomnography was used to diagnose OSA, whereas the apnea-hypopnea index (AHI) was used to assess its severity. Four studies [17-20] reported a mean AHI value (11.8 ± 13.4-39 ± 25 events/h). Four studies [17,18,20,21] reported OSA grading, according to the AHI values, representing a total of 3,076 participants. We observed mild (AHI 5 to <15 events/h), moderate (AHI 15 to 30 events/h), and severe (AHI >30 events/h) OSA cases in 1,911 (62.12%), 733 (23.82%), and 432 (14.04%) patients.

The Epworth sleepiness scale (ESS) was used to assess daytime sleepiness. Of the three literatures assessed, the overall mean ESS score ranged from 5.2 to 10.3. Shah et al. [20] reported that the ESS score was not significantly correlated with PAD. Schahab et al. [18] mentioned that the mean oxygen desaturation index (ODI) ranged from 18.8/h to 26.7/h, which showed a significant correlation with the severity (p < 0.001, Spearman’s rho = 0.91). A significant correlation was also observed between daytime sleepiness and AHI (p = 0.003, Spearman’s rho = 0.47).

Three studies [19,20,24] have reported ODI measured by polysomnography in 163 patients with OSA, which ranged from 8.9 ± 14.2 to 23 ± 10.2 events/h. However, in studies that evaluated the severity of OSA by AHI and ODI, none have mentioned the possible correlation between AHI and ODI (Table 2). Shah et al. [20] calculated the probability for a person suffering from OSA to have concurrent PAD. Following adjustment of several variables such as age, sex, BMI, smoking, and comorbidities (i.e., hypertension, coronary heart disease (CHD), diabetes, and dyslipidemia), our results indicated that patient having moderate-to-severe OSA had a 1.67 (95% CI 1.10-2.51) greater risk of PAD than those with none-to-mild OSA.

**Table 2:** Polysomnography results N: number; OSA: obstructive sleep apnea; NA: not available; PAD: peripheral arterial disease

Studies	N (number of subjects)	Overall OSA prevalence	Mean apnea-hypopnea index	Mean Epworth sleepiness scale score	Mean oxygen desaturation index (events/h)
Vogel et al., 2012 [17]	52	98% (n = 51)	39 ± 25	10.3 ± 5.4	NA
Schahab et al., 2016 [18]	59	60.4% (n = 29)	23.5 ± 16.5	NA	20.9 ± 19.1
Pizarro et al., 2014 [19]	91	70% (n = 64)	11.8 ± 13.4	NA	8.9 ± 14.2
Shah et al., 2015 [20]	8367	35% (n = 2926)	PAD: 8.63 Control: 11.14	NA	NA
Utriainen et al., 2013 [21]	82	85% (n = 70)	NA	5.2	23 ± 11.2
Nagayoshi et al., 2016 [22]	5365	3.4% (n = 182)	NA	NA	NA
Chen et al., 2015 [23]	47268	0.6%	NA	NA	NA

PAD

PAD was present in 12,207 (19.91%) patients with OSA. Ankle-brachial index (ABI), pulse wave velocity (PWV), and duplex ultrasonography were used to determine the significance of atherosclerotic plaques on regional blood flow. All the included studies described that PAD patients had an increased OSA prevalence. Vogel and colleagues [17] reported that an increase in AHI severity was significantly related to an aggravation of central PWV (p < 0.05). However, data regarding the association between AHI severity and intermittent claudication were antagonistic. Vogel et al. [17] also described a remarkable decrease in PWV with an increase in apnea severity as reflected by the AHI (p = 0.01). Contrarily, we did not observe any remarkable difference in regard to PWV among AHI groups. Utriainen et al. [21] evaluated the potential association between ABI and OSA severity measured by AHI but were unsuccessful in finding any significant association.

Discussion

Based on our existing knowledge and literature search, this study might be the first systematic review assessing the relationship between OSA and PAD. Clinically, OSA is frequent and characterized by repetitive, incomplete, or complete pharyngeal airway occlusion while sleeping, thus producing cessation of oronasal airflow. The associations of OSA with various cardiovascular conditions such as hypertension, CAD, arrhythmias, heart failure, and cerebrovascular stroke have been well documented [24]. The plausible mechanisms to be held responsible for the progression of OSA to various cardiovascular conditions, such as systemic and pulmonary hypertension, arrhythmias, stroke, and essentially to PAD, are most likely repetitious occurrences of hypoxemia and hypercapnia, which unduly promote inflammations and oxidative stress, thus leading to endothelial dysfunctions [25].

Some studies have concluded a positive correlation between OSA and PAD is not concrete. The causal relationship between OSA and PAD needs to be elucidated, and findings regarding the same should be authenticated. Other studies showed an association between OSA and increased carotid artery atherosclerosis, and appropriate treatment could reverse the observed carotid artery changes. PAD is reported to affect nearly 8.5 million Americans aged ≥40 years, resulting in several symptoms affecting patients' quality of life, such as severe claudication. PAD has also been proposed as a marker for determining the severity of atherosclerosis and shared many risk factors such as OSA, such as obesity, hypertension, diabetes, etc. [26]. Nagayoshi and colleagues [22] reported that OSA was significantly associated with PAD following an investigation on 5,365 African American patients, which persisted despite adjusting confounding factors such as age, diabetes, and sex. People who slept for shorter and longer durations were reported to have a greater risk of developing PAD. Similarly, Chen and colleagues [23] reported that moderate-to-severe OSA had an increased risk of PAD in patients matched for age and sex (odd ratio (OR) = 1.60, p < 0.001). Although such association was observed with significance following adjustment of baseline confounding factors such as age, sex, and BMI (adjusted OR = 1.37, p = 0.014), it attenuated further adjustment for confounders such as hypertension, hyperlipidemia, and DM (adjusted OR = 1.26, p = 0.075). Shah et al. [20] reported a significant correlation between OSA and patients with severe PAD (OR = 1.67, 95% CI = 1.10-2.51, p = 0.0152) but only for those with an AHI ≥15/h, which was unaffected by age, sex, hypertension, diabetes, CHD, dyslipidemia, smoking, and alcohol intake.

Continuous positive airway pressure (CPAP) remains the preferential treatment for OSA as it can significantly alleviate systemic inflammations, reduce cholesterol levels, decrease daytime sleepiness, and improve patients' life quality [27]. A previous systematic review revealed significant improvements in inflammatory markers' levels, such as C-reactive protein, tumor necrosis factor, and interleukin-6 following CPAP [28]. However, coagulation abnormalities have been persistent in patients even on prolonged CPAP therapy, which has failed to delay atherosclerotic plaque formation progression. On the contrary, some documented beneficial effects of CPAP in CAD patients in reducing cardiovascular morbidity and mortality [29]. However, few recent randomized controlled trials failed to verify CPAP benefits in reducing long-term cardiovascular outcomes regarding myocardial infarction, stroke, atrial fibrillation, etc. [30]. Hence, further investigations with stronger evidence are needed to confirm the potential effects of CPAP therapy on OSA and implications for PAD risk.

The observational design of the included studies fails to provide substantial data for a causal association of patients' characteristics. In addition, no underlying association between PAD and atherosclerosis in vascular beds other than the peripheral ones, as described in the included studies. Considering a lack of homogeneity in the reported clinical assessments, more studies are required to clarify the association between OSA and PAD. Further, the association of OSA with REM sleep has emerged as an important prognostic marker for individuals with cardiac comorbidities. Therefore, researchers should also focus on identifying the exact roles of OSA in the progression of cardiovascular complications. Understanding the pathogenesis of OSA can help alleviate these complications and improve overall health. Moreover, early diagnosis of OSA can improve the patient's prognosis.

Limitations

Our study has some strict limitations. The observational design of the majority of included studies limits the inference of any casual association for their patient data. On the one hand, our flexible exclusion criteria provide an opportunity to assess OSA prevalence in patients with isolated PAD without significant comorbidities, but on the other hand, the heterogeneous group with various existing comorbidities may overestimate that prevalence. The lack of homogeneity of association in the results of all the clinical tests for OSA and PAD warrants further investigation.

## Conclusions

Even though we described a direct link between the two diseases, they do not exist simultaneously with respect to the clinical variables used to evaluate OSA and PAD. However, AHI closely depicts the OSA severity and its association with PAD. Furthermore, ODI is regarded to truly represent the severity of OSA with PAD risk. Future studies are needed to clarify the relationship between OSA and its underlying pathophysiological association with atherosclerosis in other vascular beds. In primary care facilities, most patients presenting with OSA might suffer from undiagnosed PAD. Appropriate diagnosis at an early stage may help improve associated complications. The role of primary care physicians is vital for diagnosing PAD in patients with OSA and initiating appropriate treatment.
